# Liposome-Imipramine Blue Inhibits Sonic Hedgehog Medulloblastoma In Vivo

**DOI:** 10.3390/cancers13061220

**Published:** 2021-03-11

**Authors:** Tobey J. MacDonald, Jingbo Liu, Bing Yu, Anshu Malhotra, Jenny Munson, Jaekeun C. Park, Kenty Wang, Baowei Fei, Ravi Bellamkonda, Jack Arbiser

**Affiliations:** 1 Department of Pediatrics, Emory University School of Medicine, Atlanta, GA 30329, USA; jliu22@emory.edu (J.L.); bing.yu@emory.edu (B.Y.); anshu.malhotra@emory.edu (A.M.); 2 Aflac Cancer & Blood Disorders Center, Children’s Healthcare of Atlanta, Atlanta, GA 30329, USA; 3 Fralin Biomedical Research Institute, Room 1210, 4 Riverside Circle, Roanoke, VA 24016, USA; jennymunson@vt.edu; 4 Department of Radiology, Emory University School of Medicine, Center for Systems Imaging, Wesley Woods Health Center, 1841 Clifton Road NE, Atlanta, GA 30329, USA; jcpark2@emory.edu; 5 Department of Radiology and Imaging Sciences, Emory University School of Medicine, 1841 Clifton Rd NE, Atlanta, GA 30329, USA; kenty.wang#@emory.edu (K.W.); baowei.fei@emory.edu (B.F.); 6 Ravi V Bellamkonda Pratt School of Engineering, Duke University, Durham, NC 27708, USA; ravi@duke.edu; 7 Department of Dermatology, Emory University School of Medicine, Atlanta, GA, 30322, USA; 8 Veterans Affairs Medical Center, Decatur, GA 30322, USA

**Keywords:** medulloblastoma, reactive oxygen, Nox4, sonic hedghog

## Abstract

**Simple Summary:**

Imipramine blue (IB) is a novel NADPH oxidase inhibitor. We assessed the single agent activity of IB against a well-established model of medulloblastoma, the most common malignant brain tumor of childhood. IB slowed progression of medulloblastoma and increased survival of mice with transgenic medulloblastoma. Clinical trials of IB for medulloblastoma should be pursued.

**Abstract:**

Sonic hedgehog subtype of medulloblastoma (SHH MB) with metastasis or specific clinical or molecular alteration shas a poor prognosis and current therapy results in long-term cognitive impairment in the majority of survivors. Thus, a great need exists for new targeted therapeutic approaches to more effectively treat SHH MB in children. Imipramine blue (IB), a novel molecule with anti-tumor properties, inhibits the NADPH oxidase (NOX) family of enzymes, which are critical for SHH MB survival and treatment resistance. In this study, IB was encapsulated within a liposome to form a liposomal nanoparticle, Liposome-IB (Lipo-IB). This complex has the ability to cross the blood–brain barrier and be preferentially taken up by tumor cells within the brain. We demonstrated in vitro that Lipo-IB treatment caused a dose-dependent decrease in SHH MB cell viability and migration. Short-term administration of single agent Lipo-IB treatment of SHH MB in vivo significantly inhibited tumor growth, reduced the tumor volume, including a complete tumor response, and improved survival compared to control treated mice, without any observable toxicity. We conclude that Lipo-IB is a potential novel nanoparticle-based therapeutic for the treatment of SHH MB that warrants further preclinical safety and efficacy testing for development towards clinical investigation.

## 1. Introduction

Medulloblastoma (MB) is the most common malignant brain tumor of children, arising in the cerebellum and affecting around 1:150,000 children with a peak incidence at 5 years of age [1]. Current therapy, which combines surgical resection, craniospinal radiation and multiagent chemotherapy, offers a 5-year survival rate of >70% for newly diagnosed patients [2]. However, the success of this treatment comes at great cost, with the majority of survivors suffering skeletal growth retardation, endocrine dysfunction, psychiatric and social difficulties, and progressive cognitive impairment [3]. Furthermore, the sonic hedgehog (SHH) subtype in older children has an intermediate prognosis overall, but SHH MB patients with tumors exhibiting either metastasis, post-operative residual disease, large-cell anaplastic histology, or MYCN amplification, continue to do very poorly and are considered high to very high risk [4]. Novel MB subgroup-targeting therapeutic strategies are, thus, critical.

Another major hurdle in the successful implementation of novel therapeutics for brain tumors is the delivery of these therapeutics across the blood–brain barrier (BBB) and into brain tumor cells. In recent years, nanoparticle-based therapeutic platforms have demonstrated promise in delivering novel drugs directly to the cells within the tumor [5]. This not only makes the drug exert its full impact, but also minimizes toxic side effects to the developing brain. Therefore, novel therapies like nanoparticle-based targeted delivery of new anti-cancer drugs have drawn considerable attention in recent years. 

Recent studies from our colleagues have shown significantly increased levels of reactive oxygen species (ROS) in Sonic Hedgehog-Driven Cerebellar Progenitor Cells, which are thought to be the precursors of medulloblastoma as well as enhanced expression of NADPH oxidase 4 (NOX4) in these precursor cells [6]. An additional study demonstrated high level expression of Nox4 in medulloblastoma. A mining of the R2 public database (R2: Genomics Analysis and Visualization Platform (http://r2.amc.nl, accessed on 2 January 2020)) revealed increased expression of several NOX family enzymes across all subgroups of MB, although the highest expression was that of NOX4 (Tumor Medulloblastoma—Cavalli—763—rma_sketch—hugene11t public). This database was derived from annotation of genes discovered in the landmark paper by Cavalli et al. [7]. These findings suggest that SHH MB may be particularly vulnerable to treatment with NOX inhibitors.

Our group made the discovery that Imipramine blue (IB), a novel NOX family inhibitor that can cross the BBB, induces cell death and promotes chemosensitivity in a ROS-dependent mechanism across multiple cancer types [8,9,10,11,12]. Moreover, we previously showed that IB can be incorporated into liposomal nanocarriers to enhance brain tumor cell permeability and drug retention in a glioma model, resulting in anti-invasion effects in vivo, and increased survival when combined with chemotherapy, but not as a single agent [12]. In our current study, we demonstrate the efficacy of our liposome encapsulated IB (Lipo-IB) treatment when used as a single agent for treatment of a transgenic mouse model of SHH MB, Smoothened A1 (SmoA1) [13]. Given the imperative need for alternative targeted treatment strategies for SHH MB, we conclude that Lipo-IB is a potential novel drug and delivery method for this disease.

## 2. Results

### 2.1. Lipo-IB Treatment Inhibits SHH Medulloblastoma Viability

**Figure 1:**
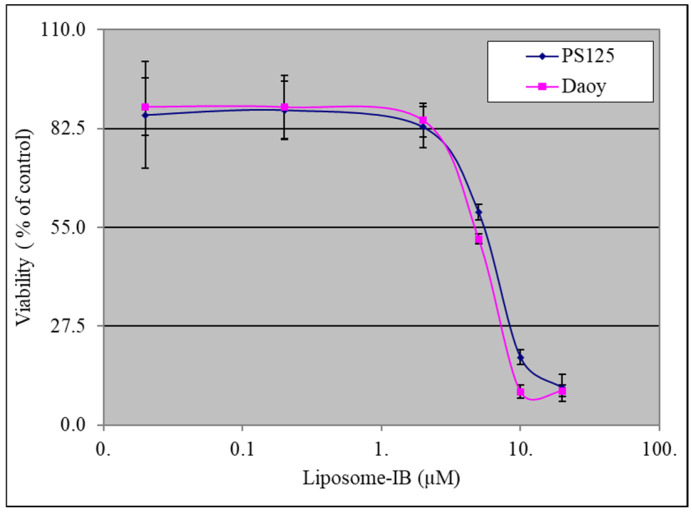
Lipo-IB inhibits sonic hedgehog (SHH) medulloblastoma cell growth. IC50 values of SmoA1 murine PS125 and human Daoy cells were calculated based on MTT assay. The data represent the mean (± standard deviation, SD) of three independent experiments, each performed in triplicate.

Given the relatively higher level of NOX4 expression in SHH MB compared to the other MB subgroups, we chose to focus on Shh-activated MB cells for testing Lipo-IB due to the potential clinical implications. To test whether Lipo-IB has an effect on Shh-activated MB cell viability, we performed a 3-(4,5-dimethylthiazol-2-yl)-2,5-diphenyl-2H-tetrazolium bromide (MTT) assay 40 h after Lipo-IB treatment of murine PS125 and human Daoy SHH MB cells. We have observed that liposomes alone have no effect on MB cells in keeping with our prior investigations using tumor cells [10,11,12]. Results show that Lipo-IB significantly inhibited SHH MB cell viability in a dose-dependent manner, with a 50% inhibitory concentration (IC50) of approximately 5.9 uM for PS125 and 5.1 uM for Daoy cells (Figure 1). This is in keeping with the IC50 range reported for non-liposomal IB (0.16–7.7 uM) against a variety of cancers [10,11,12].

### 2.2. IB Treatment Inhibits ERK Phosphorylation, p21PAK and Catalase

**Figure 2:**
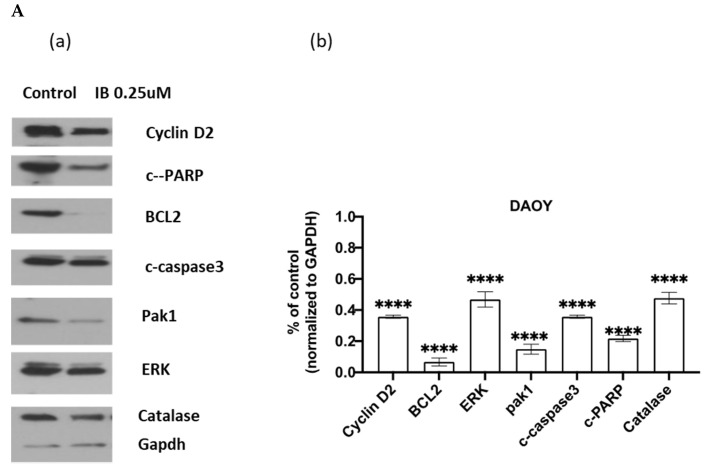

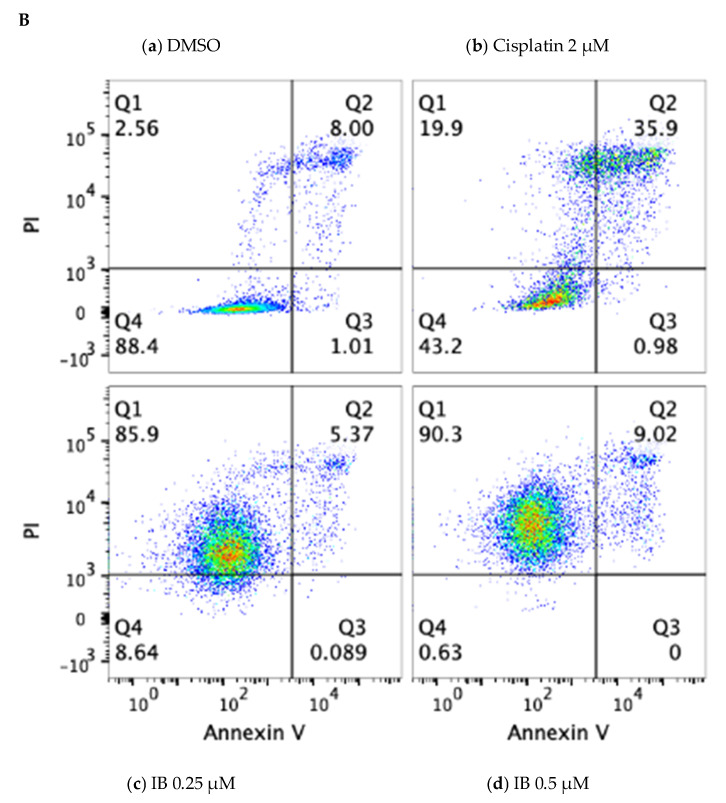
Imipramine blue decreases markers of invasive behavior and reactive oxygen signaling in nanomolar concentrations. Pak1 and ERK are markers. of invasive behavior in medulloblastoma, and catalase expression is modulated by reactive oxygen. The lack of effect of imipramine blue on PARP cleavage suggests that imipramine blue causes necrosis rather than apoptosis. (**Aa**) Daoy cells were cultured in EMEM medium containing 10% serum for 24 h, then exposed to imipramine blue (IB) 0.25 µM or vehicle for 48 h. Cell lysates were harvested for Western blot (original blots can be found at Figure S1). (**Ab**) Densitometric presentation of Western blot of three different experiments and normalized by GAPDH. Graphpad Prism 9.0 software was used to determine statistical significance between control and IB treatment, and wo-tailed Student’s *t*-test was used to assess *p* value. Error bar: mean with SD. All **** *p* value < 0.0001. B: Representative data on apoptosis profiles of Daoy cells after 48 hrs of drug treatments. IB potently induced necrosis. (**Ba**) Dimethyl sulfoxide (DMSO) treatment was used as vehicle control, while (**Bb**) Cisplatin treatment was used as a positive control for apoptosis. (**Bc**) IB treatment at 0.25 µM and (**Bd**) IB treatment at 0.5 µM. Q4: live; Q3: early apoptosis; Q2: late apoptosis; Q1: Necrosis.

Erk and PAK activation are cardinal features of medulloblastoma aggressiveness and are required for cell migration and invasive behavior [14]. Treatment with nanomolar quantities of IB led to decreased ERK expression, PAK expression, and decreased expression of catalase (Figure 2a). The downregulation of catalase is a biomarker of Nox inhibition, as Nox generated hydrogen peroxide activates catalase. IB kills medulloblastoma cells at 72 h of exposure. Surprisingly, IB treatment did not upregulate PARP cleavage, suggesting that necrosis, rather than apoptosis is the major mode of cell death. We further confirmed that IB causes necrosis through flow cytometric analysis (Figure 2b).

### 2.3. Lipo-IB Treatment Causes SHH MB Tumor Regression In Vivo and Significantly Delayed Tumor Progression in SmoA1 Mice

Based on the results from in vitro experiments, we next wanted to test the effect of Lipo-IB in vivo. Therefore, we conducted a preclinical study using the well-established transgenic SmoA1 mouse model of SHH MB, and evaluated the effect of Lipo-IB treatment on confirmed tumor-bearing mice. The primary objective here was to determine if SHH MB tumor growth could be inhibited and/or tumor progression could be delayed in SmoA1 mice with established tumors treated with Lipo-IB. 

**Figure 3:**
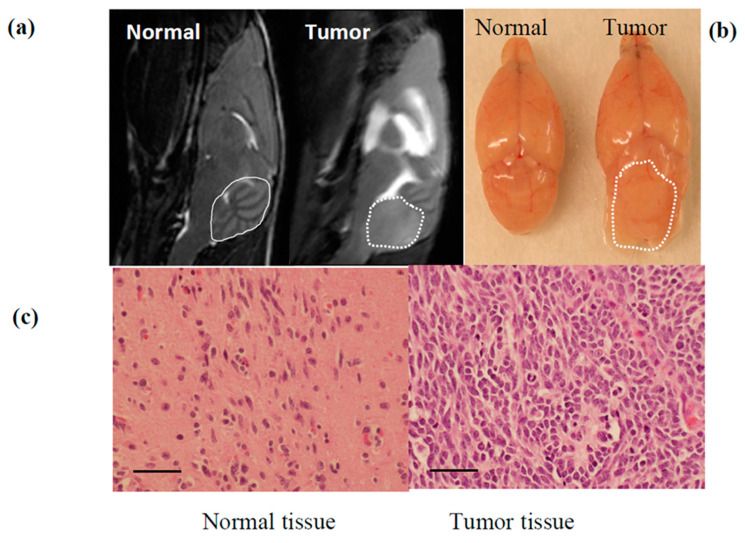
Pilot experiment to confirm MB in SmoA1 mouse is identified by MRI. (**a**) MRI scan of 12 week old SmoA1 mice demonstrating a normal brain (entire cerebellum circled in solid line) compared to mouse with MB in cerebellum (tumor circled in dash line) allowing for tumor volumes measurement prior to treatment (**b**) Dissected brain tissue showing the gross presence of MB tumor in the cerebellum (circled). (**c**) Histological evaluation by H&E straining confirming cellular architecture and confluence of sheets of small round blue tumor cells. Scale bar: 50 µM.

First, we conducted a pilot serial MR imaging (MRI) study of SmoA1 mice to determine the timing and pattern of tumor formation and growth by imaging, and found that early detection of MB could be made between 10–18 weeks of age [14]. Figure 3a shows a T2 weighted MRI image in which a tumor was detected in the cerebellum of a SmoA1 mouse at 12 weeks. We also dissected out the whole brain to confirm the presence of MB by gross and histopathological examination in all mice observed to have tumor in the cerebellum by MRI in our pilot study (Figure 3b). Corresponding histological evaluation by H&E stain verified the presence of MB (Figure 3c), identical in appearance to what has been previously published for this model [13]. Based on the results of the pilot MRI study, all experimental treatment SmoA1 cohorts were imaged at 12–15 weeks of age for the confirmation and measurement of established tumor by MRI prior to initiating treatment. No statistically significant difference was recorded between the body weight of mice and their tumor volume between the Lipo-IB treated and control groups at the time of start of treatment. The average body weight of treated mice was 22.17 g and that of controls was 23.43 g (*p* = 0.44). The median tumor volume recorded was 24.65 mm^3^ in treatment group and 41.32 mm^3^ in controls (*p* = 0.19). 

**Figure 4:**
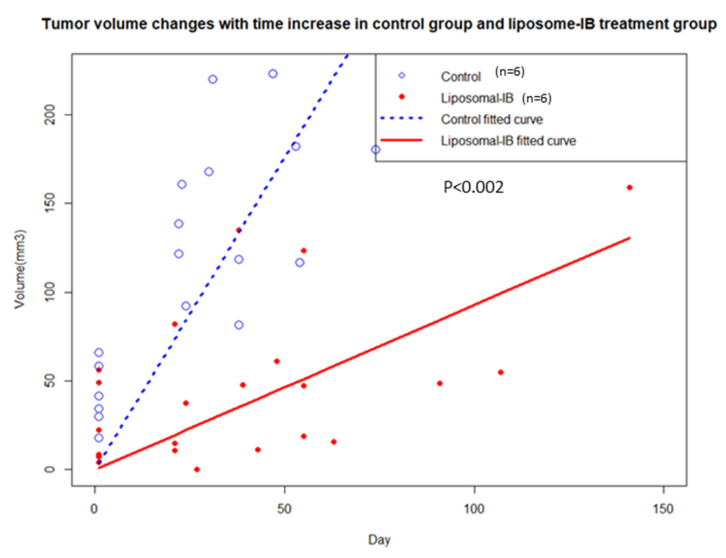
Tumor volume comparison between control and Lipo-IB treated mice for all the mice tested. Blue hollow circles (O) represent mice from control group and the red solid circles represent mice from the treatment group.

**Figure 5:**
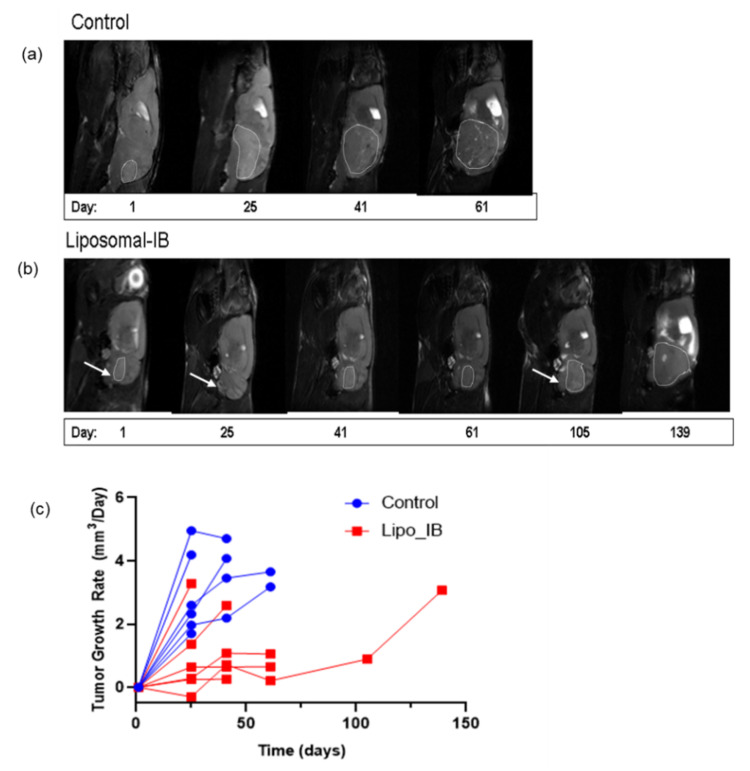
Lipo-IB treatment leads to tumor regression and significantly delays tumor progression in SmoA1 mouse. Representative results from a single mouse. (**a**) MRI scan of a tumor bearing mouse (tumor circled) at the start of the study (12 weeks old, Day 1) demonstrating typical tumor progression by serial MRI until sacrifice at Day 61 due to tumor burden. (**b**) MRI scan of a tumor bearing mouse treated with Lipo-IB. White arrows at Days 1 and 25 show reduction in tumor volume following Lipo-IB treatment. Arrow at 105 shows recurrence of tumor after discontinuation of Lipo-IB treatment. (**c**) Measurement days of tumor growth rate (GR) over time. GR = 0 represent non-growth tumor, GR < 0 represent tumor regression, GR > 0 represent tumor growth and more rapidly growing tumors have higher GR values (black arrow indicate Lipo-IB treatment start).

No gross toxicities (e.g., weight loss, diminished activity, general appearance) were observed in Lipo-IB treated mice. All the mice (treated or control) were monitored by serial MRI (T2 weighted image) to noninvasively measure change in tumor volume over time (every 2–3 weeks) in response to the Lipo-IB therapy. Tumor size increased dramatically in control group compared with the treatment group (Figure 4 and Figure 5). Images from one representative mouse from the treatment and control groups each is shown (Figure 5a,b). This mouse in the Lipo-IB treatment group, displayed complete abrogation of the tumor; however, the tumor recurred after the treatment ended one month later (Figure 5b, white arrows). The treatment efficacy in vivo was established by determining the tumor growth rate (GR), which is the relative change in tumor volume per unit time (one day was used as the minimal unit of time). We plotted the individual growth curves of all tumors, and this demonstrated significant slowing of tumor growth as a result of treatment. There were no significant differences in tumor size at the start of the experiment (Figure 5c).

### 2.4. Lipo-IB Treatment Increases SmoA1 Mice Survival

**Figure 6:**
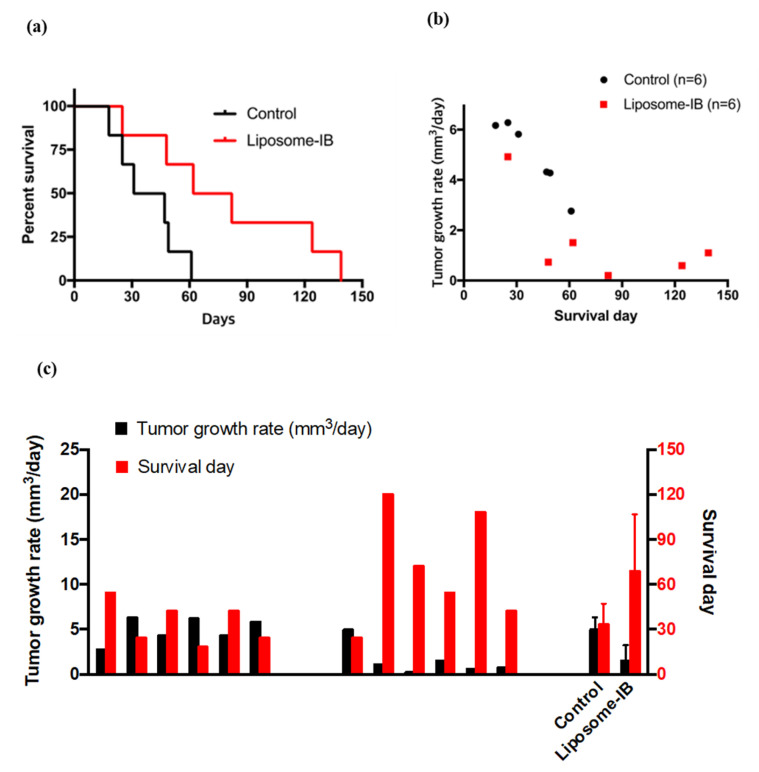
Analysis of Lipo-IB induced survival advantage. (**a**) Kaplan^=Meier curve shows significantly increased survival of Lipo-IB treated SmoA1 mice compared to control treated mice. (**b**) Pearson correlation analysis between mouse survival time and the tumor growth rate. Pearson correlation coefficient was −0.78 (*p* < 0.02). (**c**) Relationship between tumor growth rate and survival time for individual mice.

The inhibition of tumor growth in mice treated with Lipo-IB translated into an important survival advantage. The survival probability was calculated by Kaplan–Meier analysis and the survival curve is shown in Figure 6a. The result demonstrated that the survival time was significantly prolonged in the Lipo-IB treatment group. 60% of mice survived after 3 months, thus exhibiting a median survival of 82 days (95% CI:39–125), while in the control group, the median survival was 25 days (*p* < 0.05, 95% CI: 0–55). 

The Pearson correlation analysis demonstrated a significant negative correlation between the mouse survival time and the tumor specific growth rate. The Pearson correlation coefficient was −0.78 (*p* < 0.002). The relationship is shown in Figure 6b for both control and Lipo-IB treatment mice. These results indicate that longer survival time in the treatment mice was accompanied by smaller tumor specific growth rate and shorter survival time in the control mice went along with larger tumor specific growth rate. This analysis was plotted for each individual mouse in Figure 6c.

## 3. Discussion

The survival rate for SHH MB in older children with tumors displaying either metastasis, post-operative residual disease, large cell anaplastic histology, or MYCN amplification following standard treatment with radiation and chemotherapy is very poor [4], with death occurring as a result of recurrence and metastasis. Recent studies have uncovered the relationship of ROS and its associated pathways that help to enhance radiation resistance and possible recurrence of tumors [15,16,17,18,19]. NOX enzymes are a major source of ROS in most cellular environments [15,16,17,18,19]. We, and others, have previously shown the mechanism of IB in inhibiting NOX enzymes, resulting in impaired downstream signaling through Stat3, MAPK, HIF1, and TGF, as well as its efficacy against various cancers, and its ability when packaged with liposomes (Lipo-IB) to target tumor cells within the brain to prevent invasion and enhance chemosensitivity of glioma [12]. Therefore, in view of the recent discoveries about the critical role of ROS in SHH MB [7], we decided to evaluate the efficacy of Lipo-IB in SHH MB. 

Targeted therapies for MB lag behind the recent gains in molecular knowledge. Initial treatment for all MB subtypes primarily includes surgical resection followed by radiation and the use of classic chemotherapeutic agents, including alkylating agents [20]. The long-term toxicities of craniospinal radiation on growth and cognitive development are not only severe, but are also associated with an increased rate of secondary malignancy. Targeted inhibition of Shh signaling has been attempted in SHH MB with variable results, which may depend on the nature of activation of Shh in MB and the development of treatment resistance [21,22]. Our preclinical data suggest that SHH MB relies on both Shh signaling and ROS [6,7,23] and the failure to target ROS may explain the resistance and common lack of efficacy of Shh inhibition alone. The ability demonstrated by Lipo-IB in this study to inhibit the viability and migration of MB cells is significant. 

Another major hurdle often encountered during pre-clinical therapeutics’ development is the inability of most of these molecules or complexes to cross the BBB and/or effectively be taken up by tumor cells. IB was developed by coupling imipramine, which has the ability to cross the BBB, with bis (diethylamino) benzophenone in order to make a lipophilic molecule (Lipo-IB) for enhanced CNS penetration and cellular uptake [11,12]. We previously showed that Lipo-IB blocks the invasion of glioblastoma multiforme into the brain parenchyma in vivo, but requires combination with chemotherapy to achieve long term survival in rats [12]. In the current study, we demonstrate the highly robust single agent efficacy of Lipo-IB in the SmoA1 model of SHH MB in vivo, indicating SHH MB is particularly vulnerable to this NOX inhibitor. This was anticipated based on our colleagues’ prior demonstration of the critical dependency on ROS and increased NOX expression and activity in the SmoA1 model [6,7]. Nox activation results in activation of catalase as part of the detoxification mechanism of hydrogen peroxide generated by Nox-derived superoxide. In addition, we demonstrate downregulation of Erk/p21PAK activation, which is associated with an aggressive and invasive phenotype [14]. We have previously demonstrated that IB inhibition of NOX can result in down regulation of NFkB and AKT and the inhibition of p53 oxidation resulting in p53 activation [24]. Interestingly, tumoral NOX4 has also been shown to recruit M2 tumor-associated macrophages via ROS signaling to promote cancer growth, suggesting that NOX inhibitors could also have the potential to induce indirect anti-cancer effects by modulating the tumor immune microenvironment [25]. One limitation of this study is the lack of histologic evaluation for examining mechanistic changes in the tumor and immune microenvironment. Given that Lipo-IB may have direct and indirect anti-tumor effects, future studies of Lipo-IB comparing effects in immunocompetent and immunodeficient mouse MB models would help to separate the impact of the direct, and indirect, if any, anti-tumor effects we observed. Although our sample size is relatively small, the observation of complete tumor regression provides clear evidence for the activity of Lipo-IB against SHH MB. The ability of Lipo-IB to target SHH MB is a major step towards future combination studies designed to enhance the effects of chemotherapy in MB while minimizing side-effects. Previous studies have demonstrated that the BBB is not disrupted and is intact in SHH MB and the SmoA1 model [26]. Therefore, the exceptional ability of Lipo-IB to penetrate the BBB and target SHH MB tumor cells makes it an attractive effective therapeutic for SHH MB. 

In this study, we have observed potent single agent anti-tumor activity of Lipo-IB in a validated transgenic model of SHH MB. Further investigation of Lipo-IB as monotherapy, or in combination with Shh inhibition and other chemotherapeutics or biological agents is warranted. Given the devastating and life-long side-effects of brain irradiation, especially in pediatric age groups, Lipo-IB in conjunction with lower doses of irradiation and chemotherapy could provide an important alternative therapeutic strategy that is much needed for the treatment of SHH MB with high-risk features.

## 4. Materials and Methods

### 4.1. Cell Cultures 

Human Shh-activated MB cell line Daoy was obtained from American Type Culture Collection and was grown and maintained in DMEM (Biowhitaker, walkersville, MD, USA) supplemented with 10% fetal bovine serum (FBS) at 37 °C with 5% CO_2_. Murine primary Shh-activated MB cells PS125 were derived from the SmoA1 transgenic mouse model of MB, kindly provided by Dr. Robert C. Castellino, Emory University [27]. Cultures were maintained in DMEM/F-12 medium supplemented with 20% FBS as described above. 

### 4.2. Imipramine Blue and Liposome Materials 

Imipramine blue (IB) was obtained from the Arbiter laboratory of Emory University. Distearoyl phosphotidylcholine (DSPC), Poly(ethylene glycol) 2000-distearoyl phosphatidyl ethanoloamine (DSPE-PEG2000) were purchased from Avanti (Alabaster, AL, USA). Cholesterol was purchased from Sigma (St. Louis, MO, USA). Sephadex column was purchased from GE healthcare and nuclepore filters were purchased from Millipore. 

### 4.3. Nanoparticle Liposomal-IB Synthesis 

Liposomal-IB was synthesized as previously described by us, demonstrating nanoparticle drug equivalence and dose uniformity [12]. In brief, liposomes were made from DSPC (85 mol %), DSPE-PEG2000 (5 mol %) and cholesterol (10 mol %) by dissolving the lipids and 2 mg/mL of IB in ethanol. The solution was hydrated using phosphate buffered saline at 70C to yield liposomes. The liposomes were then extruded to a size of 160 nm as assessed by dynamic light scattering. Unbound drug was removed via sepharose column separation and then diafiltrated to a final IB concentration of 1.7 mg/mL.

### 4.4. Western Blot

Daoy was cultured in DMEM medium containing 10% serum for 24 h, then added imipramine blue 0.25 µM for 48 h. Whole cell lysates were harvested in lysis buffer (Cell Signaling Technology, Danvers, MA) for Western Blot. Western blot was performed with the following primary antibodies: PAK1, Catalase, ERK1/2, Cyclin D2 and Gapdh (Cell Signaling Technology, Danvers, MA); BCL2, Cleaved -PARP and Caspase-3(ABCAM, Cambridge, MA, USA). Goat anti-rabbit antibodies (Santa Cruz, Dallas, TX, USA) were used and the immunoreactive bands were detected by ECL. Original images can be found at Figure S1.

### 4.5. Flow Cytometry

For cell apoptosis profiling, treated Daoy cells were stained with Alexa Fluor 488 annexin V/Dead Cell Apoptosis kit (Fisher Scientific, Waltham, MA, USA). Results were acquired on a BD FACSymphony™ A5 Cell Analyzers (BD Biosciences, San Jose, CA, USA) and analyzed with FlowJo 10 (Tree Star, Inc., Ashland, OR, USA).

### 4.6. Cell Viability Assay

The effect of Lipo-IB on cell viability was analyzed by MTT assay Cell Proliferation Kit I (Roche Applied Science, Indianapolis, IN, USA). Briefly, PS125(2.0 × 10^4^ cells/well) and Daoy (1.0 × 10^4^ cells/well) cells were seeded into the wells of a 96-well plate, in 3 replicates and allowed to grow overnight. Approximately 18 h later, they were treated with Lipo-IB at different concentrations (0, 0.01, 0.05, 0.1, 0.2, 1.0 µM). 40 h after the addition of Lipo-IB, MTT assay was performed following the manufacturer’s protocol. Independent experiments were performed three times, each in triplicate.

### 4.7. Animal Model and Treatment

SmoA1 transgenic mice (C57BL/6-Tg (Neurod2-Smo*A1)199 Jols/J) were purchased from the Jackson Laboratory. All animal procedures were conducted in accordance with the Guidelines for the Care and Use of Laboratory Animals and were approved by the Institutional Animal Care and Use Committee at Emory University (IACUC DAR-2003564-083016N). A total of 14 mice were screened for tumor formation by MRI at 12–15 weeks of age. This was done to ensure that tumors were detected at an early stage so that the effects of Lipo-IB could be observed for a longer duration of time, until euthanasia became inevitable. Mice were screened periodically every month until tumor formation was detected. The mice with early tumors confirmed by MRI were randomized to receive tail vein injections of Lipo-IB (4 mg/kg; *n* = 6), which was administered in 2 doses at 5-day intervals each, respectively. The in vivo dose was previously established by us in a glioma model [12] and treatment was discontinued after only two doses. A control group of mice (*n* = 6) received similar injections of liposomal control in parallel. 

### 4.8. Magnetic Resonance Imaging (MRI) and Tumor Volume Measurement

Each SmoA1 mouse was anesthetized by 1–2% isoflurane, and then placed in a 9.4T MRI. MRI measurements were performed using a 9.4 T/20 cm horizontal bare Bruker magnet, interfaced to an Avance console (Bruker, Billerica, MA). A two-coil actively decoupled imaging set-up was used (a 2 cm diameter surface coil for reception and a 7.2 cm diameter volume coil for transmission). Sagittal T2-weighted images were acquired with a RARE (Rapid Acquisition with Refocused Echoes) sequence. Imaging parameters were as follows: repetition time (TR) = 4000 ms, effective Echo (Eff. TE) = 48 ms, RARE factor = 8, field of view (FOV) = 20 × 20 mm^2^, matrix = 116 × 116, Aug = 12, slice thickness (thk) = 0.75 mm, number of slice = 15, 4 average per phase encode step requiring a total acquisition time of about 25 min per mouse. Tumor volume was quantified as the products of slice-to-slice separation and the sum of areas from the manually draw tumor ROI on images. 

### 4.9. Statistical Analysis

Determination of statistical significance was assessed by student’s *t*-test for the in vitro studies and ANOVA and Kaplan–Meier for the in vivo studies using the IBM SPSS Statistics software.

## 5. Conclusions

Imipramine blue is a novel NADPH oxidase inhibitor. It has previously shown synergistic activity against glioblastoma with doxorubicin. In this paper, we demonstrate single agent activity of IB against Sonic hedgehog mediated medulloblastoma as a single agent. IB decreases pro-invasive MAPK signaling in human medulloblastoma cells, and induces necrosis, rather than apoptosis. In vivo, IB was highly effective in prolonging survival in mice that develop medulloblastoma through the Sonic hedgehog pathway. This study provides justification to pursue clinical trials on the Sonic hedgehog subset of pediatric medulloblastomas, which are an orphan indication.

## Data Availability

The data presented in this study are available in this article (and supplementary material).

## Supplementary Materials

The following are available online at https://www.mdpi.com/2072-6694/13/6/1220/s1, Figure S1: The original Western blots.
