# Supplementary material for: Liposome-Imipramine Blue Inhibits Sonic Hedgehog Medulloblastoma In Vivo
Source: Cancers (Basel). 2021 Mar 11;13(6):1220. doi: 10.3390/cancers13061220 (PMC8001973; doi:10.3390/cancers13061220)
Supplement: Supplementary file 1 [file cancers-13-01220-s001.pdf]

Supplementary Materials

Liposome-Imipramine Blue Inhibits Sonic Hedgehog Medulloblastoma In Vivo

Tobey J. MacDonald, Jingbo Liu, Bing Yu, Anshu Malhotra, Jenny Munson, Jaekeun C. Park, Kenty Wang, Baowei Fei, Ravi Bellamkonda, Jack Arbiser

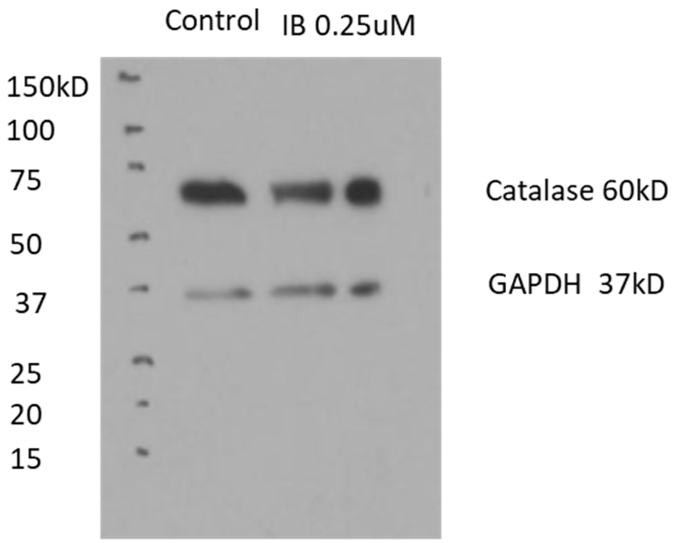

Catalase and GAPDH

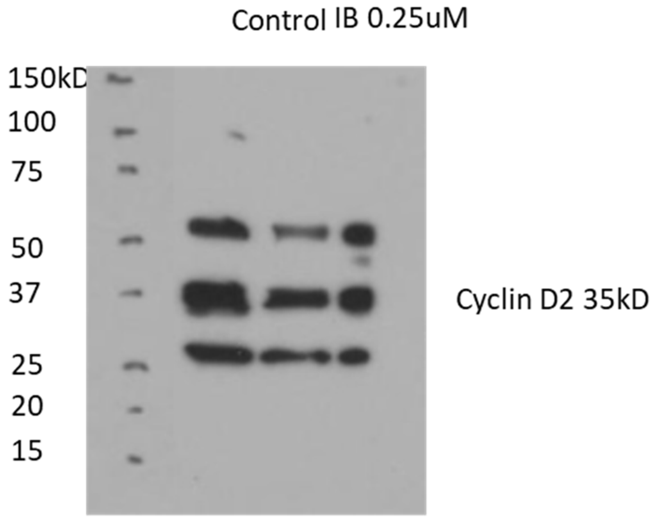

Cyclin D2

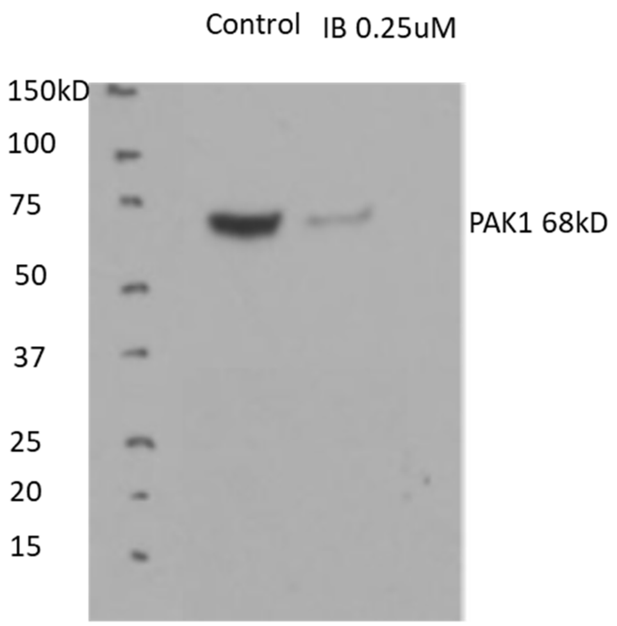
Control
IB 0.25uM
150kD
100
75
50
37
25
20
15
PAK1 68kD

PAK1

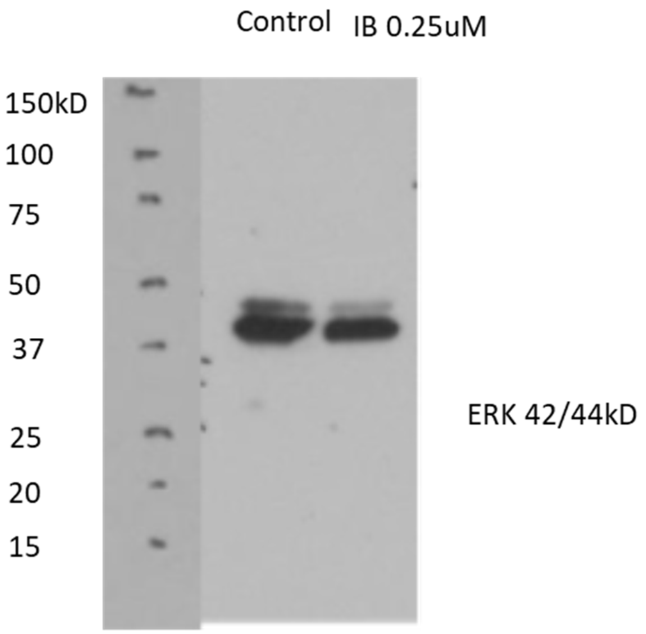
Control
IB 0.25uM
150kD
100
75
50
37
25
20
15
ERK 42/44kD

ERK

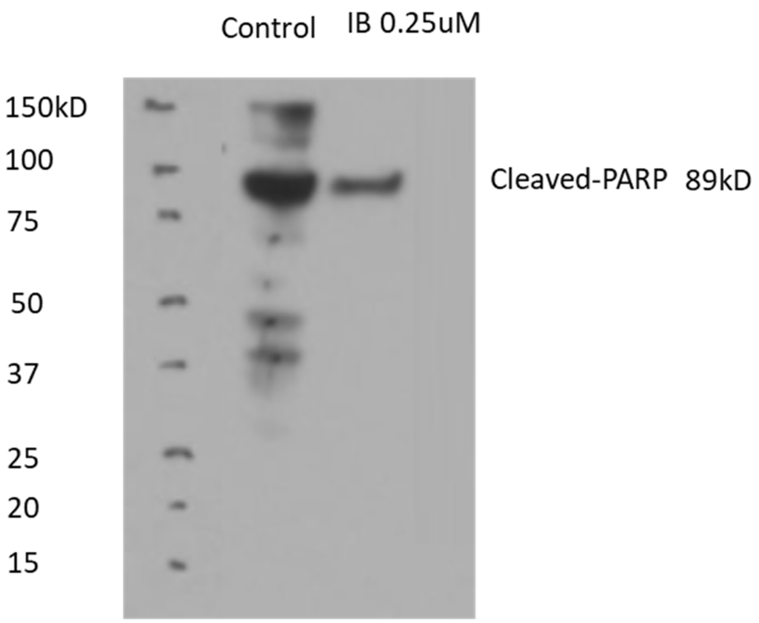

Cleaved-PARP

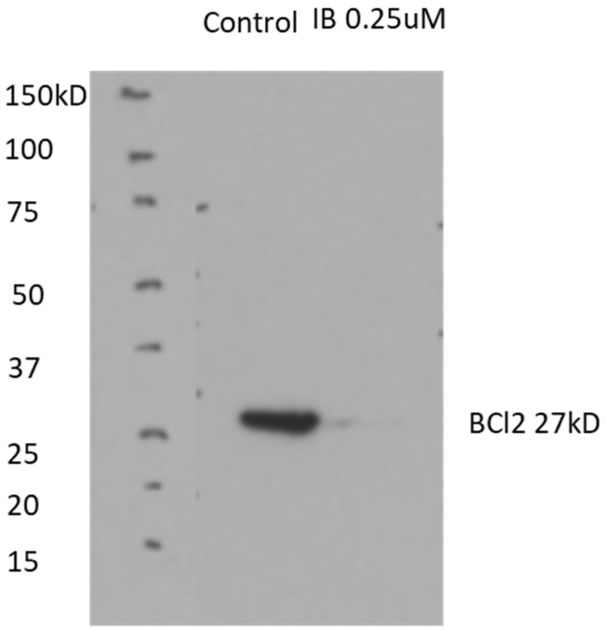

BCL2

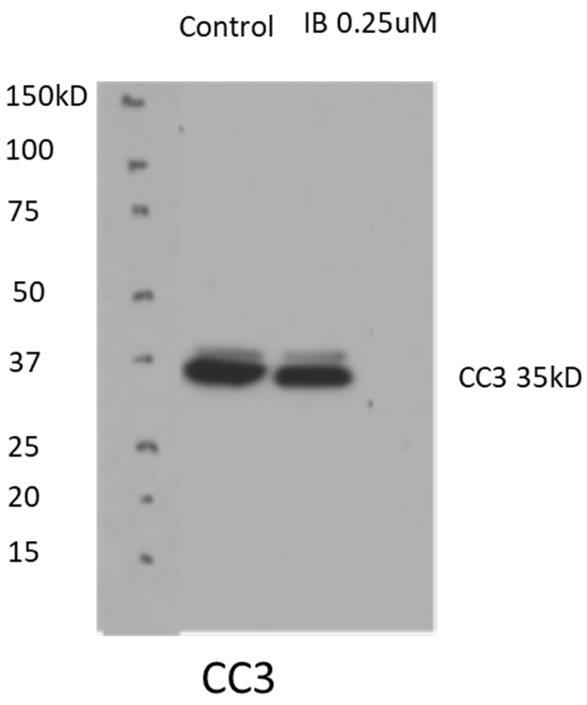

Figure S1. Original Western Blots.
